# Mechanisms on chemotherapy resistance of colorectal cancer stem cells and research progress of reverse transformation: A mini-review

**DOI:** 10.3389/fmed.2022.995882

**Published:** 2022-09-12

**Authors:** Lei Chen, Funing Yang, Si Chen, Jiandong Tai

**Affiliations:** ^1^Department of Colorectal and Anal Surgery, General Surgery Center, First Hospital of Jilin University, Changchun, China; ^2^Pediatric Outpatient Clinic, First Hospital of Jilin University, Changchun, China

**Keywords:** colorectal cancer, cancer stem cell, chemotherapy resistance, stem cells, reverse

## Abstract

Tumor recurrence and chemotherapy resistance are mainly responsible for poor prognosis in colorectal cancer (CRC) patients. Cancer stem cell (CSC) has been identified in many solid tumors, including CRC. Additionally, CSC cannot be completely killed during chemotherapy and develops resistance to chemotherapeutic drugs, which is the main reason for tumor recurrence. This study reviews the main mechanisms of CSC chemotherapy resistance in CRC, including activation of DNA damage checkpoints, epithelial-mesenchymal transition (EMT), inhibition of the overexpression of antiapoptotic regulatory factors, overexpression of ATP-binding cassette (ABC) transporters, maintenance of reactive oxygen species (ROS) levels, and the dormant state of CSC. Advances in research to reverse chemotherapy resistance are also discussed. Our study can provide the promising potential for eliminating CSC and preventing tumor progression for CRC treatment.

## Introduction

**Figure 1:**
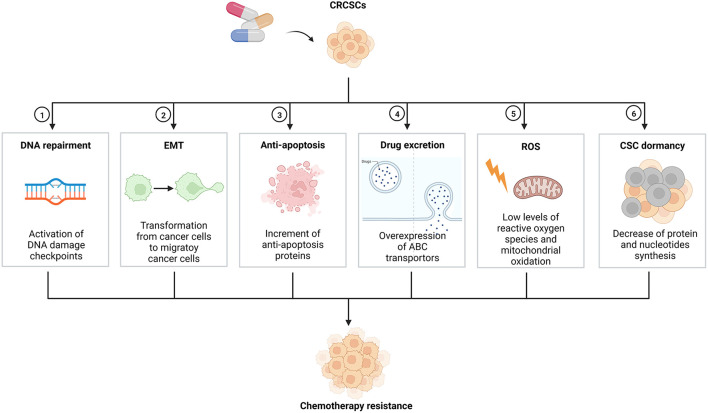
Mechanisms on chemotherapy resistance of CRCSCs. CRCSCs, colorectal cancer stem cells; EMT, epithelial-mesenchymal transition; ABC, ATP-binding cassette; ROS, reactive oxygen species; CSC, cancer stem cell.

Colorectal cancer (CRC) is a worldwide disease, with 2.2 million CRC patients and 1.1 million deaths expected by 2030 (1, 2). Additionally, CRC is the most common malignant tumor of the lower digestive tract, with distinct genetic, epigenetic and phenotypic heterogeneity of tumor cells (3). Despite rapid advances in diagnosis methods, surgery, and chemotherapeutic agents, the prognosis of CRC patients remains poor (4). Tumor recurrence and cancer chemotherapy resistance are leading causes of poor prognosis (5). Inhibition of tumor apoptosis, changes in targeted sites of chemotherapeutic agents, tumor cell heterotrophy, and cancer stem cells (CSCs) can lead to chemotherapy resistance, while CSCs are the key factor for chemotherapy resistance (6). Accounting for about 5% of total tumor cells, CSC is a special cell population capable of self-renewal, multi-lineage differentiation, cloning, tumor initiation, maintenance of tumor characteristics, metastasis, and proliferation (7). In addition, CSC has been identified in various cancers, including breast, colorectal, pancreatic, lung, prostate and brain cancers (8, 9). Chemotherapy resistance in the CRC stem cell (CRCSC) have diverse mechanisms (Figure 1), mainly including activation of DNA damage checkpoints, epithelial-mesenchymal transition (EMT), inhibition of the overexpression of antiapoptotic regulatory factors, overexpression of ATP-binding cassette (ABC) transporters, and maintenance of reactive oxygen species (ROS) levels. In recent years, more and more researchers have focused on natural drug extracts and CSC-related inhibitors, which can effectively remove CSC and reverse chemotherapy resistance (10). This study reviews the mechanism and the reversal of CRCSC chemotherapy resistance.

## Mechanisms of CRCSC chemotherapy resistance

### Activation of DNA damage checkpoints

CSC can activate DNA damage checkpoints preferentially in response to DNA damage caused by DNA toxic drugs, thereby improving DNA repair. For example, CD133^+^ hepatic stem cells preferentially express survival proteins related to the Akt/PKB and Bcl-2 pathways, thereby leading to the chemotherapy resistance of cancer cells to adriamycin and 5-fluorouracil (5-FU) (11, 12). Additionally, DNA damage response may act as a target for sensitizing CSC to overcome chemotherapy resistance (13). Another study suggested that 70% of ovarian cancer patients developed relapse and resistance after platinum-based chemotherapy (14). Methoxyphenyl chalcone can play a role in DNA damage signal-evoking potential that can reverse the chemotherapy resistance. Moreira et al. found that induction of DNA double-strand breaks can effectively kill CSCs, which is vital for overcoming multiple conventional chemotherapy resistance in CRC (15).

### EMT

The association between EMT and chemotherapy resistance has been discussed for a long time, but the mechanism is still elusive. Some researchers hold the opinion that cells undergo EMT process have a stem-cell like property, thus sharing the key signaling pathways and drug resistance characteristics with CSC (16, 17). Other important mechanisms related to EMT-induced drug resistance mainly include the gain of cellular resistance to drug-driven apoptosis (18) and associated tumor microenvironment (19). For example, cancer-associated fibroblasts and hypoxia can activate the EMT process of cancer cells and induced drug resistance (18). EMT plays an important role in epigenetic changes in CRC cells and is also associated with the self-renewal ability, tumor heterogeneity, and chemotherapy resistance of CSC. A study showed that hyaluronic acid synthases led to the loss of epithelial traits in tumor cells, which further induced malignant tumors and created a suitable niche for CSC generation (20). The study indicated the fundamental role of EMT in tumor progression and chemotherapy resistance of CSC. Pathak et al. found that downregulation of the mitochondrial Na^+^/Ca^2+^/Li^+^ exchanger (NCLX) facilitated the metastasis of CRC cells, EMT changes, hypoxia, chemoresistance, and stem cell pathways (21). However, the study did not reveal a direct mechanism between EMT changes and chemoresistance of CSC.

### Inhibition of the overexpression of antiapoptotic regulatory factors

When CSC is exposed to chemotherapeutic agents *in vivo*, some special regulatory mechanisms will be activated; pi3-K, MAPK and other pathways will be cascade-activated, and then the anti-apoptotic protein myeloid cell leukemia-1 (McL-1) will increase to inhibit the apoptosis of tumor cells (22). Many studies have suggested that inhibiting the activity of CSCs can increase the apoptosis of tumor cells (23–25). In another study by Zhang et al., CRCSCs were inhibited by pitavastatin, and the apoptosis of colon carcinoma cells was increased (26). However, detailed mechanisms of CSC against the expression of antiapoptotic regulatory factors remain unknown.

### Overexpression of ABC transporters

Another potentially important mechanism of CSC leading to chemotherapy resistance is the high expression of ABC transporters. In CSC, the high expression of the ATP-dependent efflux pump ABCG2 enabled it to effectively extract Hoechst 33342, which is a kind of DNA minor groove binder used fluorochrome for visualizing cellular DNA, from cells (27, 28). This export ability is provided by the ABC transporter, which helps to resist cytotoxic drugs. The ABC transporter can export certain chemicals and drugs from cells, resulting in multidrug resistance (29). Stem-like side population tumor cells are key to the cause of drug resistance. A study indicated that the expression of ABCG2 in side population cells within tumors with stem-like properties was higher than that in non-side population tumor cells. Glucose in the microenvironment could further up-regulate the expression of ABCG2 (30). Because the high expression of ABC transporter allowed cells to effectively pump traditional chemotherapeutic drugs out of cells, the high ABCG2 level in the side population made it resistant to many chemotherapeutic drugs (30). Therefore, inhibition of ABC pump function may be a potential strategy to overcome CSC resistance. Although many ABC transporter inhibitors have been shown to sensitize cancer cells to chemotherapeutic drugs *in vitro*, their effectiveness has failed to demonstrate in most clinical trials (31).

### Maintenance of ROS levels

ROS, such as superoxide anion and hydrogen peroxide, is the product of normal oxidative metabolism and is involved in many cellular signaling processes. High ROS levels can promote cell migration and differentiation, which impairs the long-term reproduction and survival of tumor cells (32). Normally, ROS maintains at a low level in CSC by increasing glycolysis and reducing mitochondrial oxidation and ROS production (33, 34). Chemotherapy resistance caused by overexpression of multidrug resistance molecules can be overcome by inhibiting glycolytic consumption of cellular ATP (35). Low ROS levels in CSC can support its survival; conversely, excessively high ROS levels may trigger the death of CSC (36). The low ROS level in CSC is partly due to the high expression of free radical scavenger molecules such as glutathione (37). Glutathione participates in cell detoxification by binding to toxic chemicals and certain chemotherapeutic agents (such as cisplatin) and facilitating their export from cells (38, 39). A recent study indicated that intravenous vitamin C combined with traditional cancer treatment significantly decelerated cancer progression (40). Vitamin C promoted oxidation by increasing intracellular ROS levels, inducing endoplasmic reticulum stress, and inhibiting the production of angiogenic factors and insulin-like growth factors. As a result, high-dose vitamin C alone or in combination with chemotherapy (e.g., paclitaxel, cisplatin, carboplatin, and azacytidine) may increase ROS levels in CRCSC and CRC to inhibit tumor.

### The dormant state of CSC

Another factor contributing to the drug resistance of CSC is the quiescent or dormant state (41, 42). A metabonomics analysis of CRC showed a significantly down-regulated synthesis of protein in colo205 CD133^+^ CRC cells compared with CD133 cells and the reduced synthesis of nucleotides such as cholesterol and glucose-dependent lipid (43). The unique metabolic characteristics of CRCSC exhibit a slow circulation property that leads to chemotherapy resistance. Because many chemotherapeutic drugs preferentially kill fast-growing cells, tumor cells are more active in DNA replication and are highly sensitive to DNA damage agents. In contrast, relatively dormant CRCSCs are unlikely to induce non-replicating functional DNA in non-circulating cells, so they are insensitive to DNA damage agents (44). Moreover, CSC will have more time to repair DNA damage and stay alive. Therefore, even though most circulating tumor cells can be killed by chemotherapy, residual CSCs can enter the cell cycle and cause tumor recurrence (45).

## The research progress of reverse transformation

### Natural agents

Curcumin, a plant polyphenol, is the most important component of ginger (46). Recent studies have not only proved the effect of turmeric in traditional Chinese medicine but also suggested some new pharmacological effects, such as anti-inflammatory, antioxidant, oxygen free radical scavenging, anti-human immunodeficiency virus, liver and kidney protection, anti-fibrosis and anti-cancer effects (47). In recent years, the chemotherapy resistance reverse effect of curcumin in CRC has attracted increasing attention. Kanwar et al. confirmed that curcumin combined with traditional chemotherapeutic agents 5-FU and oxaliplatin reduced the expression of CD44 and CD166 in chemo-resistant colon cancer cells, inhibited tumor growth, and promoted apoptosis in tumor tissue (48). Detailed results of mechanisms revealed that curcumin combined with 5-FU and oxaliplatin could prevent the growth of CSC-enriched chemo-resistant CRC cells by inhibiting epidermal growth factor receptor (EGFR) and insulin-like growth factor 1 receptor (IGF-1R) signaling pathways. Curcumin has been proved to enhance the sensitivity of drug-resistant CRC cells to many traditional chemotherapeutic agents by eliminating CSC (49, 50). Another study demonstrated that curcumin could enhance the chemotherapy efficacy of 5-FU on HCT116 cells, indicating that curcumin may help to treat CRC and overcome chemotherapy resistance (51). Further insights into the mechanism demonstrated that curcumin could inhibit multiple CSC pathways, suggesting its anti-CSC potential in CRC treatment (49).

Salvianolic acid B (SALB), a water-soluble phenolic compound extracted from Salvia miltiorrhiza, can reverse chemotherapy resistance and improve the clinical treatment effect of CRC. Guo et al. developed a nude mouse model bearing human colon CSCs and investigated the effect of SALB on chemotherapy resistance reversal and related mechanisms (52). The nude mice were transplanted with LoVo and HCT-116 colon CSCs to establish an animal model that could exhibit chemotherapy resistance. The results revealed that SALB reversed chemotherapy resistance to 5-FU and oxaliplatin and inhibited tumor growth by suppressing the expression of stemness markers, such as CD44, CD133, and the transcription factor sox-2 (SOX2). In addition, SALB has been proven to target CSCs *in vitro* and *in vivo* and prevent tumor progression by modulating the IL-6/STAT3/NF-κB signaling pathway (53).

Aloysia polystachya (AP) is a medicinal plant extract widely used to treat various diseases. Additionally, CSC is highly associated with tumor invasiveness, chemotherapy resistance and cell death. It was found that AP significantly reduced the invasiveness of HCT116 and CT26 cell lines and the number of tumorspheres compared with the control group (54). When HCT116 and CT26 cells were treated with 5-FU and AP, their sensitivity to low concentrations of 5-FU was increased by AP. These results suggested that the inhibition effect of AP on CSC might be one of the mechanisms to reverse 5-FU resistance.

### Inhibitors

Regorafenib is an approved specific multikinase receptor inhibitor for the treatment of metastatic CRC. Cai et al. developed two 5-FU resistance CRC cell lines, HCT-116R and DLD-1R, to evaluate regorafenib inhibition of CRCSCs (55). Combined with 5-FU, regorafenib suppressed tumorigenesis and stemness markers in DLD-1R cell lines. Moreover, regorafenib increased the miR-34a levels and induced the reverse transformation of drug resistance. In another study, researchers implanted human colon cancer cells KM12SM and mesenchymal stem cells (MSCs) into the cecal wall of nude mice, which could provide tumors with abundant stromal components and improve invasion and metastasis ability and drug resistance (56). The results indicated that regorafenib could affect the interaction of tumor cell-MSCs and further inhibit CRC progression.

## Conclusion

CSC has been found in many solid tumors, such as CRC, breast cancer, pancreatic cancer, and lung cancer, and is considered a promising target for cancer treatment (7, 8). It cannot be completely killed during chemotherapy and develops resistance to chemotherapeutic drugs, which is mainly responsible for tumor recurrence, metastasis and poor prognosis. This study reviews the main mechanisms of CSC chemotherapy resistance in CRC, including activation of DNA damage checkpoints, EMT, inhibition of the overexpression of antiapoptotic regulatory factors, overexpression of ABC transporters, maintenance of ROS levels, and the dormant state of CSC. Natural plant extracts (e.g., curcumin, SALB, and AP) and specific multikinase receptors (e.g., regorafenib) exhibit promising potential in eliminating CSC and preventing tumor progression.

More work is needed for CRCSC chemotherapy reverse transformation. First, researchers should find more specific markers to distinguish CSC from normal stem cells because CSC shares molecular similarities with embryonic stem cells and MSCs, which limits the potential for targeted therapy. Second, more research should be performed to significantly improve the delivery efficiency of effective drugs to targeted cells and reduce the side effects of chemotherapy, which provides a new direction for targeted therapy of CRC. Third, more natural drugs and their extracts should be studied to screen out the best natural drug, drug dosage and delivery mode and use in the modern treatment of CRC patients. Fourth, studies on inhibitors of related enzymes for treating CRC should receive more attention. Fifth, more animal and clinical studies should be performed to provide a theoretical basis for the reverse transformation of chemotherapy resistance of CRCSC using specific natural agents and inhibitors.

## Author contributions

LC drafted the review. FY and SC generated the graph and guided the construction of the manuscript. JT edited the review. All the authors contributed to the article and approved the submitted version.

## Funding

This study was supported by Financial Department of Jilin Province (JLSCZT2019-018).

## Conflict of interest

The authors declare that the research was conducted in the absence of any commercial or financial relationships that could be construed as a potential conflict of interest.

## Publisher's note

All claims expressed in this article are solely those of the authors and do not necessarily represent those of their affiliated organizations, or those of the publisher, the editors and the reviewers. Any product that may be evaluated in this article, or claim that may be made by its manufacturer, is not guaranteed or endorsed by the publisher.

## References

[1] Elbadawy M Usui T Yamawaki H Sasaki K. Development of an experimental model for analyzing drug resistance in colorectal cancer. Cancers. (2018) 10:164. 10.3390/cancers10060164 29843359 PMC6025190

[2] Miller KD Nogueira L Devasia T Mariotto AB Yabroff KR Jemal A. Cancer treatment and survivorship statistics, 2022. Can J Clin. (2022). 10.3322/caac.21731 35736631

[3] Garza Trevino EN Delgado Gonzalez P Valencia Salgado CI Martinez Garza A. Effects of pericytes and colon cancer stem cells in the tumor microenvironment. Can Cell Int. (2019) 19:173. 10.1186/s12935-019-0888-9 31303863 PMC6604392

[4] Biller LH Schrag D. Diagnosis and treatment of metastatic colorectal cancer: a review. JAMA. (2021) 325:669–85. 10.1001/jama.2021.0106 33591350

[5] Voutilainen S Heikkila P Sampo M Nevanlinna H Blomqvist C Mattson J. Expression of markers of stem cell characteristics, epithelial-mesenchymal transition, basal-like phenotype, proliferation, and androgen receptor in metaplastic breast cancer and their prognostic impact. Acta Oncol. (2021) 60:1233–9. 10.1080/0284186x.2021.1950927 34282709

[6] Tang D Yang Z Long F Luo L Yang B Zhu R. Long noncoding rna malat1 mediates stem cell-like properties in human colorectal cancer cells by regulating mir-20b-5p/Oct4 Axis. J Cell Physiol. (2019) 234:20816–28. 10.1002/jcp.28687 31012108

[7] Zhou H-M Zhang J-G Zhang X Li Q. Targeting cancer stem cells for reversing therapy resistance: mechanism, signaling, and prospective agents. Signal Trans Targeted Therapy. (2021) 6:62. 10.1038/s41392-020-00430-1 33589595 PMC7884707

[8] Duan H Liu Y Gao Z Huang W. Recent advances in drug delivery systems for targeting cancer stem cells. Acta Pharmaceutica Sinica B. (2021) 11:55–70. 10.1016/j.apsb.2020.09.016 33532180 PMC7838023

[9] Yang L Shi P Zhao G Xu J Peng W Zhang J. Targeting cancer stem cell pathways for cancer therapy. Signal Trans Targeted Therapy. (2020) 5:8. 10.1038/s41392-020-0110-5 32296030 PMC7005297

[10] Weng W Goel A. Curcumin and colorectal cancer: an update and current perspective on this natural medicine. Semin Cancer Biol. (2022) 80:73–86. 10.1016/j.semcancer.2020.02.011 32088363 PMC7438305

[11] Behrooz AB Syahir A Ahmad S. Cd133: beyond a cancer stem cell biomarker. J Drug Target. (2019) 27:257–69. 10.1080/1061186x.2018.1479756 29911902

[12] Tsunekuni K Konno M Haraguchi N Koseki J Asai A Matsuoka K. Cd44/Cd133-positive colorectal cancer stem cells are sensitive to trifluridine exposure. Sci Rep. (2019) 9:14861. 10.1038/s41598-019-50968-6 31619711 PMC6795793

[13] Ronco C Martin AR Demange L Benhida R. Atm, Atr, Chk1, Chk2 and Wee1 Inhibitors in Cancer and Cancer Stem Cells. Medchemcomm. (2017) 8:295–319. 10.1039/c6md00439c 30108746 PMC6072143

[14] Su Y-k Huang W-C Lee W-H Bamodu OA Zucha MA Astuti I. Methoxyphenyl chalcone sensitizes aggressive epithelial cancer to cisplatin through apoptosis induction and cancer stem cell eradication. Tumor Biol. (2017) 39:1010428317691689. 10.1177/1010428317691689 28466786

[15] Moreira H Szyjka A Paliszkiewicz K Barg E. Prooxidative activity of celastrol induces apoptosis, DNA damage, and cell cycle arrest in drug-resistant human colon cancer cells. Oxidative Med Cell Long. (2019) 2019:6793957. 10.1155/2019/6793957 31485297 PMC6710751

[16] Biddle A Liang X Gammon L Fazil B Harper LJ Emich H. Cancer stem cells in squamous cell carcinoma switch between two distinct phenotypes that are preferentially migratory or proliferative. Cancer Res. (2011) 71:5317–26. 10.1158/0008-5472.Can-11-1059 21685475

[17] Liu S Cong Y Wang D Sun Y Deng L Liu Y. Breast cancer stem cells transition between epithelial and mesenchymal states reflective of their normal counterparts. Stem Cell Reports. (2014) 2:78–91. 10.1016/j.stemcr.2013.11.009 24511467 PMC3916760

[18] Du B Shim JS. Targeting epithelial-mesenchymal transition (Emt) to overcome drug resistance in cancer. Molecules. (2016) 21:965. 10.3390/molecules21070965 27455225 PMC6273543

[19] Bremnes RM Donnem T Al-Saad S Al-Shibli K Andersen S Sirera R. The role of tumor stroma in cancer progression and prognosis emphasis on carcinoma-associated fibroblasts and non-small cell lung cancer. J Thoracic Oncol. (2011) 6:209–17. 10.1097/JTO.0b013e3181f8a1bd 21107292

[20] Nguyet N Kumar A Chacko S Ouellette RJ Ghosh A. Human hyaluronic acid synthase-1 promotes malignant transformation via epithelial-to-mesenchymal transition, micronucleation and centrosome abnormalities. Cell Commun Sign. (2017) 15:48. 10.1186/s12964-017-0204-z 29137675 PMC5686803

[21] Pathak T Gueguinou M Walter V Delierneux C Johnson MT Zhang X. Dichotomous role of the human mitochondrial Na+/Ca2+/Li+ exchanger nclx in colorectal cancer growth and metastasis. Elife. (2020) 9:e59686. 10.7554/eLife.59686 32914752 PMC7529464

[22] Cammareri P Scopelliti A Todaro M Eterno V Francescangeli F Moyer MP. Aurora-a is essential for the tumorigenic capacity and chemoresistance of colorectal cancer stem cells. Cancer Res. (2010) 70:4655–65. 10.1158/0008-5472.Can-09-3953 20460511

[23] Cai P Xiao Z Pan T Wen X Cao J Ouyang B. Lx2-32c inhibits the formation of mammosphere from Mda-Mb-231 cells and induces apoptosis involving in down-regulating Foxm1. Biomed Pharmacother. (2018) 102:1176–81. 10.1016/j.biopha.2018.03.143 29710535

[24] Ko Y-C Choi HS Liu R Kim J-H Kim S-L Yun B-S. Inhibitory effects of tangeretin, a citrus peel-derived flavonoid, on breast cancer stem cell formation through suppression of Stat3 signaling. Molecules. (2020) 25:2599. 10.3390/molecules25112599 32503228 PMC7321155

[25] Tian Y Song Y Bai W Ma X Ren Z. Cxcr4 knockdown inhibits the growth and invasion of nasopharyngeal cancer stem cells. Oncol Lett. (2017) 13:2253–9. 10.3892/ol.2017.5694 28454388 PMC5403420

[26] Zhang ZY Zheng SH Yang WG Yang C Yuan WT. Targeting colon cancer stem cells with novel blood cholesterol drug pitavastatin. Eur Rev Med Pharmacol Sci. (2017) 21:1226–33. 28387909

[27] Wu C Alman BA. Side population cells in human cancers. Cancer Lett. (2008) 268:1–9. 10.1016/j.canlet.2008.03.048 18487012

[28] Ho MM Ng AV Lam S Hung JY. Side population in human lung cancer cell lines and tumors is enriched with stem-like cancer cells. Cancer Res. (2007) 67:4827–33. 10.1158/0008-5472.Can-06-3557 17510412

[29] Guo Q Grimmig T Gonzalez G Giobbie-Hurder A Berg G Carr N. Atp-binding cassette member B5 (Abcb5) promotes tumor cell invasiveness in human colorectal cancer. J Biol Chem. (2018) 293:11166–78. 10.1074/jbc.RA118.003187 29789423 PMC6052213

[30] Liu PP Liao J Tang ZJ Wu WJ Yang J Zeng ZL. Metabolic regulation of cancer cell side population by glucose through activation of the akt pathway. Cell Death Differ. (2014) 21:124–35. 10.1038/cdd.2013.131 24096870 PMC3857620

[31] Yu M Ocana A Tannock IF. Reversal of atp-binding cassette drug transporter activity to modulate chemoresistance: why has it failed to provide clinical benefit? Can Metast Rev. (2013) 32:211–27. 10.1007/s10555-012-9402-8 23093326

[32] Jia P Dai C Cao P Sun D Ouyang R Miao Y. The role of reactive oxygen species in tumor treatment. RSC Adv. (2020) 10:7740–50. 10.1039/c9ra10539e 35492191 PMC9049915

[33] Dayem AA Choi H-Y Kim J-H Cho S-G. Role of oxidative stress in stem, cancer, and cancer stem cells. Cancers. (2010) 2:859–84. 10.3390/cancers2020859 24281098 PMC3835109

[34] Ding S Li C Cheng N Cui X Xu X Zhou G. Redox regulation in cancer stem cells. Oxidative Med Cell Long. (2015) 2015:750798. 10.1155/2015/750798 26273424 PMC4529979

[35] Akram M. Mini-review on glycolysis and cancer. J Cancer Educ. (2013) 28:454–7. 10.1007/s13187-013-0486-9 23728993

[36] Kobayashi CI Suda T. Regulation of reactive oxygen species in stem cells and cancer stem cells. J Cell Physiol. (2012) 227:421–30. 10.1002/jcp.22764 21448925

[37] Nagano O Okazaki S Saya H. Redox regulation in stem-like cancer cells by Cd44 variant isoforms. Oncogene. (2013) 32:5191–8. 10.1038/onc.2012.638 23334333

[38] Wang J Luo B Li X Lu W Yang J Hu Y. Inhibition of cancer growth in vitro and in vivo by a novel ros-modulating agent with ability to eliminate stem-like cancer cells. Cell Death Dis. (2017) 8:e2887. 10.1038/cddis.2017.272 28640251 PMC5520927

[39] Yu L-Y Shen Y-A Chen M-H Wen Y-H Hsieh P-I Lo C-L. The feasibility of ros- and gsh-responsive micelles for treating tumor-initiating and metastatic cancer stem cells. J Mater Chem B. (2019) 7:3109–18. 10.1039/c8tb02958j

[40] Carr AC Cook J. Intravenous vitamin c for cancer therapy - identifying the current gaps in our knowledge. Front Physiol. (2018) 9:1182. 10.3389/fphys.2018.01182 30190680 PMC6115501

[41] Recasens A Munoz L. Targeting cancer cell dormancy. Trends Pharmacol Sci. (2019) 40:128–41. 10.1016/j.tips.2018.12.004 30612715

[42] Talukdar S Bhoopathi P Emdad L Das S Sarkar D Fisher PB. Dormancy and cancer stem cells: an enigma for cancer therapeutic targeting. In: Civin CI, Kingsbury TJ, Kim M, Fisher PB, editors. Cancer Stem Cells. Advances in Cancer Research (2019). p. 43–84. 10.1016/bs.acr.2018.12.002 30691685

[43] Vincent Z Urakami K Maruyama K Yamaguchi K Kusuhara M. Cd133-positive cancer stem cells from colo205 human colon adenocarcinoma cell line show resistance to chemotherapy and display a specific metabolomic profile. Genes Cancer. (2014) 5:250–60. 10.18632/genesandcancer.23 25221643 PMC4162140

[44] Eyler CE Rich JN. Survival of the fittest: cancer stem cells in therapeutic resistance and angiogenesis. J Clin Oncol. (2008) 26:2839–45. 10.1200/jco.2007.15.1829 18539962 PMC2739000

[45] Skvortsova I Debbage P Kumar V Slwortsov S. Radiation resistance: cancer stem cells (Cscs) and their enigmatic pro-survival signaling. Semin Cancer Biol. (2015) 35:39–44. 10.1016/j.semcancer.2015.09.009 26392376

[46] Esatbeyoglu T Huebbe P Ernst IMA Chin D Wagner AE Rimbach G. Curcuminufrom molecule to biological function. Angewandte Chemie Int Edition. (2012) 51:5308–32. 10.1002/anie.201107724 22566109

[47] Stohs SJ Chen O Ray SD Ji J Bucci LR Preuss HG. Highly bioavailable forms of curcumin and promising avenues for curcumin-based research and application: a review. Molecules. (2020) 25:1397. 10.3390/molecules25061397 32204372 PMC7144558

[48] Kanwar SS Yu Y Nautiyal J Patel BB Padhye S Sarkar FH. Difluorinated-curcumin (Cdf): a novel curcumin analog is a potent inhibitor of colon cancer stem-like cells. Pharm Res. (2011) 28:827–38. 10.1007/s11095-010-0336-y 21161336 PMC3792588

[49] Ramasamy TS Ayob AZ Myint HHL Thiagarajah S Amini F. Targeting colorectal cancer stem cells using curcumin and curcumin analogues: insights into the mechanism of the therapeutic efficacy. Cancer Cell Int. (2015) 15:96. 10.1186/s12935-015-0241-x 26457069 PMC4599442

[50] Elbadawy M Hayashi K Ayame H Ishihara Y Abugomaa A Shibutani M. Anti-cancer activity of amorphous curcumin preparation in patient-derived colorectal cancer organoids. Biomed Pharmacother. (2021) 142:112043. 10.1016/j.biopha.2021.112043 34411919

[51] Shakibaei M Kraehe P Popper B Shayan P Goel A Buhrmann C. Curcumin potentiates antitumor activity of 5-fluorouracil in a 3d alginate tumor microenvironment of colorectal cancer. Bmc Cancer. (2015) 15:250. 10.1186/s12885-015-1291-0 25884903 PMC4406109

[52] Guo P Wang J Gao W Liu X Wu S Wan B. Salvianolic acid B reverses multidrug resistance in nude mice bearing human colon cancer stem cells. Mol Med Rep. (2018) 18:1323–34. 10.3892/mmr.2018.9086 29845279 PMC6072146

[53] Zhao H Han B Li X Sun C Zhai Y Li M. Salvia miltiorrhiza in breast cancer treatment: a review of its phytochemistry, derivatives, nanoparticles, and potential mechanisms. Front Pharmacol. (2022) 13:872085. 10.3389/fphar.2022.872085 35600860 PMC9117704

[54] Soares Machado M Palma A Panelo LC Paz LA Rosa F Cecilia Lira M. Extract from aloysia polystachya induces the cell death of colorectal cancer stem cells. Nutr Cancer Int J. (2020) 72:1004–17. 10.1080/01635581.2019.1669676 31573355

[55] Cai M-H Xu X-G Yan S-L Sun Z Ying Y Wang B-K. Regorafenib suppresses colon tumorigenesis and the generation of drug resistant cancer stem-like cells via modulation of Mir-34a associated signaling. J Exp Clin Cancer Res. (2018) 37:151. 10.1186/s13046-018-0836-x 30005681 PMC6045878

[56] Takigawa H Kitadai Y Shinagawa K Yuge R Higashi Y Tanaka S. Multikinase inhibitor regorafenib inhibits the growth and metastasis of colon cancer with abundant stroma. Cancer Sci. (2016) 107:601–8. 10.1111/cas.12907 26865419 PMC5001714

